# The Effects of Composite Alkali-Stored Spent *Hypsizygus marmoreus* Substrate on Carcass Quality, Rumen Fermentation, and Rumen Microbial Diversity in Goats

**DOI:** 10.3390/ani14010166

**Published:** 2024-01-04

**Authors:** Shuiling Qiu, Keyao Li, Xiangbo He, Mingming Gu, Xinghui Jiang, Jianing Lu, Zhiyi Ma, Xuewu Liang, Qianfu Gan

**Affiliations:** 1 College of Animal Science (College of Bee Science), Fujian Agriculture and Forestry University, Fuzhou 350002, China; shuiling.qiu@aliyun.com (S.Q.); 3200609004@fafu.edu.cn (K.L.); fafuhxb2017@163.com (X.H.); 13763870270@163.com (M.G.); jxh0843@163.com (X.J.); lujn129@163.com (J.L.); mzy1561566@163.com (Z.M.); 2 Laboratory of Animal Nutritional Physiology and Metabolic Process, Key Laboratory of Agro-Ecological Processes in Subtropical Region, Institute of Subtropical Agriculture, Chinese Academy of Sciences, Changsha 410125, China

**Keywords:** spent mushroom substrate, alkali storage, fattening goat, carcass quality, rumen microorganism

## Abstract

**Simple Summary:**

Spent *Hypsizygus marmoreus* substrate (SHMS) is a byproduct of the *Hypsizygus marmoreus* harvest and contains numerous nutrients and active substances. It is a new type of high-quality feed material for livestock and poultry, with a certain application value. The objective of this study was to investigate the effects of composite alkali-stored SHMS on carcass quality, rumen fermentation, and rumen microbial diversity in goats. The results showed that adding it to the diet improved the carcass quality. It also increased the content of the total volatile fatty acids in rumen and had a positive impact on rumen fermentation. Furthermore, it altered the rumen microbial community structure of the goats. These research findings can provide scientific references for the utilization of SHMS as feed in the goat industry.

**Abstract:**

The objective of this study was to investigate the effects of composite alkali-stored spent *Hypsizygus marmoreus* substrate (SHMS) on carcass quality, rumen fermentation, and rumen microbial diversity in goats. Twenty-four 6-month-old Chuanzhong black goats with similar body weights (20 ± 5 kg) were selected and randomly divided into four groups (*n* = 6 per group) and received four treatments: 0% (control group, CG); 20% (low-addition group, LG); 30% (moderate-addition group, MG); and 40% (high-addition group, HG) of SHMS-replaced silage corn and oat hay. The experiment lasted for 74 days (including a 14 d adaptation period and a 60 d treatment period). The results of this study showed that MG and HG significantly improved the marble score of goat meat (*p* < 0.05). The flesh color score significantly increased in each group (*p* < 0.05). The fat color scores significantly increased in LG and MG (*p* < 0.05). There were no significant effects on the pH value or shear force of the longissimus dorsi in each group (*p* > 0.05). The cooking loss in MG was higher than that in CG (*p* < 0.05). The histidine and tyrosine contents in each group of muscles significantly increased (*p* < 0.05), with no significant effect on fatty acids (*p* > 0.05). The rumen pH of MG significantly decreased (*p* < 0.05), while the total volatile fatty acids (TVFAs) and ammoniacal nitrogen (NH_3_-N) increased by 44.63% and 54.50%, respectively. The addition of the SHMS altered both the alpha and beta diversities of the rumen microbiota and significant differences in the composition and structure of the four microbial communities. The dominant bacterial phylum in each group were Firmicutes and Bacteroidetes, with *Prevotella 1* as the dominant bacterial genus. Correlation analysis revealed that rumen bacteria are closely related to the animal carcass quality and rumen fermentation. In the PICRUSt prediction, 21 significantly different pathways were found, and the correlation network showed a positive correlation between the *Prevotella 1* and 7 metabolic pathways, while the C5-branched dibasic acid metabolism was positively correlated with nine bacteria. In summary, feeding goats with an SHMS diet can improve the carcass quality, promote rumen fermentation, and alter the microbial structure. The research results can provide a scientific reference for the utilization of SHMS as feed in the goat industry.

## 1. Introduction

In the context of the circular economy, maximizing the utilization of agricultural byproducts as substitutes for traditional feed is one of the effective strategies to alleviate feed shortages, address the issue of waste disposal, and reduce the production cost of livestock breeding [1]. The rational development and utilization of agricultural byproducts are of great significance in achieving green industrial circulation and promoting the sustainable development of animal husbandry.

*Hypsizygus marmoreus* is an industrial mushroom widely cultivated in East Asia and is popular among consumers for its unique umami flavor and various medicinal effects [2]. The dietary effect is attributed to the presence of active substances, such as polysaccharides, proteins, essential amino acids, lectins, vitamins, and enzymes, thus having potential effects on improving human health [3]. However, the byproducts after mushroom harvesting (spent *Hypsizygus marmoreus* substrate, SHMS) have not been well treated, and traditional incineration, landfilling, composting, and other treatments have caused environmental pollution and resource waste [4]. The spent mushroom substrate contains a large amount of mycelium and exhibits nutritional properties worthy of study, such as antioxidant capacity, antibacterial and antifungal activities, and anti-aging activity [5,6]. Therefore, it is a new type of high-quality unconventional feed material for livestock and poultry [7]. There have been reports on the use of the waste mushroom matrix as a livestock feed [8]. Furthermore, researchers process and store mushroom byproducts through physical, chemical, and microbial fermentation to better utilize and preserve these resources [9,10]. These studies indicate that adding the waste mushroom matrix to feeds has a positive impact on animals’ production performance and improves the nutritional composition of meat products [11].

Currently, consumers have an increasing demand for lamb and its meat products while also paying more attention to the quality and flavor of the lamb. The quality of the lamb (such as the meat color and amino acid and fatty acid contents) affects consumer choices. Nutritional regulation and feeding strategies are effective means to improve meat quality [12]. Therefore, exploring the impact of the diet on meat quality has a guiding significance for production. On the other hand, the diet affects rumen fermentation and is closely related to the production performance and meat quality of ruminants [13].

The rumen is a unique digestive organ in ruminants, housing a large number of microorganisms. The rumen microbiome interacts with the host in a symbiotic relationship, aiding in the adaptation to high-fiber plants and providing energy for the host’s growth through the fermentation of nutrients [14,15]. The dietary composition is a crucial factor affecting the rumen microbiota, and changes in these nutrients can impact the structure of the rumen microbiota community [16]. In a previous study, it was found that adding a certain proportion of the fermented spent mushroom substrate from *Pleurotus eryngii* to the diet of Hu sheep increased the diversity of the rumen microbiota and altered the microbial community’s structure [17]. However, research on the impact of the spent mushroom substrate on the rumen microbiota of ruminants is limited, and there is a lack of studies on the application of composite alkali-stored SHMS in ruminant nutrition. This study aims to investigate the effects of composite alkali-storage SHMS on goat carcass quality, rumen fermentation, and rumen microbiota in order to provide a theoretical basis for the rational development and application of SHMS.

## 2. Materials and Methods

### 2.1. Preparation of Test Materials

The substrate of *Hypsizygus marmoreus* was provided by Fujian Fuquanxin Company Co., Ltd. (Ningde, China). In this experiment, only fresh and once-picked mushroom bran without mold was selected. The fresh substrate was mixed with 5% CaO and 3% urea, respectively, based on dry matter (DM), and sealed by direct compaction. The composite alkali was stored at room temperature for 30 days. The formula for the *Hypsizygus marmoreus* medium and the nutritional components of the mushroom bran are shown in Table S1 (Supplementary Materials). Pesticide residues, heavy metal residues, and aflatoxin residues were detected by Fujian Provincial Analysis and Testing Center, Ingel Detection Technology Service Shanghai Co., Ltd., (Shanghai, China) and Qingdao Kechuang Quality Inspection Co., Ltd. (Qingdao, China). The results showed that the sanitary standard of the mushroom bran met the GB 13078-2017 feed hygiene standard [18], and it can be used for feeding (Supplementary Materials: Table S2).

### 2.2. Animals, Diets, and Feeding Management

This experiment was conducted on the farm of Fujian Ningde Qiutan Agriculture Co. (Ningde, China). Twenty-four healthy 6-month-old Chuanzhong black male goats with similar weights (20 ± 5 kg) were selected and randomly divided into 4 groups, with six goats in each group. Two individuals with similar weights are placed in the same fence (3 m × 2.5 m), and each fence is equipped with a feeding box. The control group followed the conventional diet formula, while the experimental group used compound alkali silage (5% CaO + 3% urea) to replace 20%, 30%, and 40% of the roughage (silage corn, oat hay) in the control group (CG). These replacement levels were referred to as the less addition group (LG), the moderate addition group (MG), and the high addition group (HG), respectively. Dietary nutrition levels were prepared with reference to NRC guidelines. The diet composition and nutrition levels are shown in Table 1. Except for silage corn, the remaining raw materials were made into granules. The diet was prepared as a total mixed diet (TMR) according to the experimental diet formula.

Before the experiment, the shed was cleaned and disinfected, and all experimental goats underwent infectious disease prevention and disinsection. During the testing period, the goats had free access to food and water and were fed twice daily at 8:00 and 16:00. Specially assigned personnel ensured that the enclosure remained clean and hygienic with natural ventilation. The experiment lasted for 74 days (including 14 d adaptation period and 60 d treatment period).

### 2.3. Sample Collection

At the end of the trial period, all 24 goats were slaughtered and underwent a 16 h fasting period. Additionally, they were prohibited from drinking water for 2 h prior to slaughter. Rumen contents were collected immediately after slaughter and stored at −80 °C for the determination of rumen pH, volatile fatty acids (VFAs), ammoniacal nitrogen (NH_3_-N), and rumen microbiota. Longissimus dorsi samples were collected from between the 12th and 13th ribs on both the left and right sides of the carcass. These samples were utilized for meat color scoring, pH value determination, cooking loss, and shear force analysis, as well as the assessment of amino acid, fatty acid content, and health indicators of the longissimus dorsi.

### 2.4. Carcass Quality Analysis

#### 2.4.1. Meat Color Score

The marble score, flesh color score, and fat color score were measured in the longissimus dorsi between the 12th and 13th ribs using the scoring standards of the objective evaluation method for the edible quality of national meat (China). Among them, the marble pattern level map was rated on a scale of 1 to 5, with a larger number indicating better quality (1 = almost none, 2 = a small amount, 3 = medium, 4 = rich marbling, 5 = abundant marbling). Muscle color and fat color were divided into 8 levels based on color depth (muscle color: 1 = light pink, 8 = deep red; fat color: 1 = pure white, 8 = yellow).

#### 2.4.2. Meat pH Determination

The back end of the thoracic segment of the longissimus dorsi was taken. The thickness of the meat should be at least 2.0 cm, the diameter should be at least 3.0 cm, and the mass should be at least 10.0 g. A cross-shaped incision on the tested sample was made to insert the probe and measure the pH value.

#### 2.4.3. Cooking Loss and Shear Force Determination

Weigh the longissimus dorsi sample and weigh it (M1), place it in a sealed bag, extract any air bubbles, submerge it completely in 80 °C water for 30 min, then cool it under running water at 15 °C for 40 min. Afterward, open the plastic bag, gently wipe off any surface moisture from the meat sample using filter paper, and reweigh it (M2). Cooking loss (%) = (M1 − M2)/M1 × 100%.

Shear force measurement is performed using the Warner Bratzler shear force device (TTA.XT Plus, Stable Micro Systems, Godalming, UK). Take a meat sample with a central temperature of 0~4 °C, heat it in a constant temperature water bath at 80 °C, and measure the central temperature of the meat sample using a thermocouple thermometer. Wait until the central temperature of the meat sample reaches 70 °C. Afterward, cool to 0~4 °C and perpendicularly shear the meat sample parallel to the muscle fibers. Measure immediately after sampling. Unit is expressed in Newton (N).

#### 2.4.4. Health Indicators Determination

The determination of carcass hygiene physical and chemical indicators (volatile salt base, hydrargyrum (Hg), arsenic (AS), cadmium (Cd), plumbum (Pb), chromium (Cr)) was referred to the GB2707-2016 National food safety standard—Fresh (frozen) livestock and poultry products standard [19] and sent to Qingdao Zhengxin Detection and Analysis Co., Ltd. (Qingdao, China) for testing.

#### 2.4.5. Amino Acid and Fatty Acid Determination

The amino acids and fatty acids in the longissimus dorsi were determined according to the national food safety standard “Determination of amino acids in food” [20], the content of flavored amino acids in muscle was determined using an amino acid analyzer (Hitachi L-8900, Tokyo, Japan). To begin, mix 1.50 g of chopped lamb with 15 mL of 5-sulfosalicylic acid (300 g/mL) in a centrifuge tube. Next, incubate the mixture in the dark for 1.5 h and centrifuge it in a high-speed freeze centrifuge (4 °C, 10,000 r/min, 15 min). Following this, take 2 mL of the supernatant, filter it with a 0.22 μ needle filter, and transfer it to an automatic amino acid analyzer for amino acid analysis and determination.

According to the national food safety standard “Determination of fatty acids in food” [21], the content of muscle fatty acids is determined using a gas chromatograph (Agilent-6890 N, Santa Clara, CA, USA). After thawing the sample at room temperature, separate the muscle and fat and grind them in liquid nitrogen in a mortar. Take 1 g of the sample and transfer it to a 15 mL centrifuge tube. Add 0.7 mL of 10 mol/L potassium hydroxide solution and 5.3 mL of anhydrous methanol. Place in a 55 °C water bath for 1.5 h while shaking the test tube for 5 s every 20 min. Cool to room temperature, add 0.5 mL of 12 mol/L sulfuric acid solution, maintain a constant temperature water bath at 55 °C, and shake the test tube every 20 min for 5 s and 1.5 h. Cool to room temperature, add 3 mL of n-hexane to a centrifuge tube, centrifuge at 3000 r/min for 5 min, filter the supernatant into a sample bottle, take 2 mL of the supernatant, and use 0.22 μ filter with a needle filter and conduct testing.

### 2.5. Rumen Fermentation Characteristics

Ruminal fluid pH was measured using a Remagnet PHB-4 pH meter (Shanghai Yidian Scientific Instrument Co., Ltd., Shanghai, China). Ammoniacal nitrogen (NH_3_-N) was determined according to the phenol-sodium hypochlorite colorimetric method [22].

To determine the content of acetic acid, propionic acid, butyric acid, isobutyric acid, valeric acid, and isovaleric acid in volatile fatty acids (VFAs), use a gas chromatography instrument (Agilent 6890N, Santa Clara, CA, USA; Chromatographic column: 19,091 N-133 HP-INNOWAX, 30 mm × 0.25 mm × 0.25 um). Take 2 g sample and place it into a test tube. Add a mixed solution of 5 mL hydrochloric acid (1%) and formic acid (5%) to a 1.0 mg/mL 2-ethylbutyric acid solution and shake well. Then, place it in an ice water bath for 30 min, intermittently oscillating. Afterward, remove it and centrifuge at 1500 r/min for 10 min. Transfer a certain amount to a 1.5 mL centrifuge tube and centrifuge at 14,000 r/min for 10 min. Finally, take the supernatant and measure it using a 0.45 μM membrane filtration.

### 2.6. Rumen Microbiome Analysis

In this experiment, cetyltris (cockroach) ammonium bromide lysis buffer (CTAB) combined with bead milling was used to extract total DNA from rumen microorganisms [23]. In order to analyze microbial diversity, we used universal primers 341F (5′-CCTACGGGNGGCWGCAG-3′) and 806R (5′-GGACTACHVGGGTATCTAAT-3′) to amplify the V3-V4 variable region of the 16S rRNA gene. The PCR amplification system was 50 μL, including 5 μL of 10× KOD Buffer, 5 μL of 2.5 mM dNTPs, 1.5 μL of primer (5 μM), 1 μL of KOD polymerase, and the amplification conditions were as follows: pre-denaturation at 95 °C for 2 min, followed by denaturation at 98 °C for 10 s, annealing at 62 °C for 30 s, extension at 68 °C for 30 s for 27 cycles, and finally extension at 68 °C for 10 min. PCR amplification products were recovered by gel cutting and quantified by QuantiFluor TM fluorometer (Shanghai Shengqizhao Biotechnology Co., Ltd., Shanghai, China). The purified amplification products were mixed in equal amounts, and sequencing connectors were ligated. The constructed libraries were sequenced on the Illumina Hiseq 2500 platform.

The construction, sequencing, and data analysis of the database were carried out by Guangzhou Gidio Biotechnology Co., Ltd., (Guangzhou, China). Use FASTP to read and filter the original data. FLASH [24] (v1.2.11) combines dual end reads. Use QIME [25] (v1.9.1) to obtain high-quality clean labels and remove the chimera via UCHIME algorithm. UPARS Epipeline [26] was used to cluster tags with more than 97% sequence identity into operational taxonomic units (OTUs). The RDP classifier [27] (v2.2) was used to annotate the SILVA [28] database with OTUs. The alpha and beta diversity indices were calculated using QIIME and Implement Chao1 index, abundance-based coverage estimator (ACE) index, Shannon diversity index, and Simpson index. Visualize using principal coordinate analysis (PCoA) graphs β diversity analysis; this graph is estimated based on unweighted and weighted UniFrac distance metrics. PICRUSt2 [29] (v1.0) was used to predict functional abundance.

### 2.7. Statistical Analysis

The experimental data were sorted out by Excel 2020, and SPSS 20.0 software was used for one-way analysis of variance (ANOVA). Duncan’s method was used for multiple comparisons, and *p* < 0.05 was used as the significant difference standard.

Various R packages (v4.1.3) (ggplot2, stats, linkET, corrplot) help to establish histograms and analyze PCoA, isometric matrix results of microbial composition based on OTU abundance at the phylum and genus levels, and graphical analysis of species or functional abundance spectra. Using Mantel correlation, Spearman rank correlation coefficient to test the relationship between variables.

## 3. Results

### 3.1. Changes in Carcass Quality and Health Indicators

As shown in Table 2, the marble score of the MG and HG groups showed a significant increase compared to the CG group (*p* < 0.05). The dietary intervention had a notable impact on the meat color score of the lamb, as the meat color score of each experimental group was significantly higher than that of the CG group (*p* < 0.05). Regarding the fat color score, both the MG and HG groups achieved higher scores (*p* < 0.05). There were no significant differences observed in the pH value of the dorsal longest muscle across the groups (*p* > 0.05). However, the cooking loss of the dorsal longest muscle in the MG group was significantly higher than that in the CG group (*p* < 0.05). Additionally, no significant differences were found in the shear force of the dorsal longest muscle among the groups (*p* > 0.05).

As shown in Table 3, the health and safety assessment of goat carcasses revealed the following findings: no Hg or As were detected. The levels of volatile salt base, Cd, Pb, and Cr were found to be below the standard limits, demonstrating compliance with livestock and poultry products standards. 

### 3.2. Changes in Amino Acid and Fatty Acid Fraction of Carcasses

Table 4 shows the results of the amino acid profile of the longissimus dorsi. The contents of tyrosine and histidine in the MG and HG groups were significantly higher than that in the CG group (*p* < 0.05), while there were no significant differences in other amino acids between the groups (*p* > 0.05). Additionally, as the dosage of SHMS increased, there was a significant increasing trend in the amino acid contents of EAAs, N-EAAs, and FAAs in the carcass (0.05 < *p* < 0.1).

The fatty acid profile of the longissimus dorsi is shown in Table 5. A total of eleven fatty acids were detected in the carcasses of fattened goats, including five saturated fatty acids (SFAs), four monounsaturated fatty acids (MUFAs), and two polyunsaturated fatty acids (PUFAs). The contents of palmitic acid (C16:0) and stearic acid (C18:0) in SFA are relatively high. PUFA mainly consists of linoleic acid (C18:2n6). There were no significant differences in the fatty acid and carcass characteristics among the experimental groups (*p* > 0.05).

### 3.3. Variation of Rumen Fermentation Parameters

Adding SHMS affects rumen fermentation (Table 6). In volatile fatty acids (VFAs), the content of acetic acid was the highest, reaching 62.02% to 64.32%, but there was no significant difference among the experimental groups (*p* > 0.05). The content of propionic acid in CG was significantly higher than that in the other three groups (*p* < 0.05). The contents of butyric acid and isovaleric acid in LG were significantly higher than those in the other three groups (*p* < 0.05). The isobutyric acid content of HG was significantly higher than that of CG, but there was no significant difference between LG and MG (*p* > 0.05). The total volatile fatty acids (TVFAs) and NH_3_-N of MG were significantly higher than those of CG (*p* < 0.05), and there was a trend of improvement in LG and MG compared with CG, but the difference was not significant (*p* > 0.05); the pH of rumen fluid of MG was significantly lower than that of CG and other test groups (*p* < 0.05).

### 3.4. Diversity of the Rumen Microbiome

**Figure 1:**
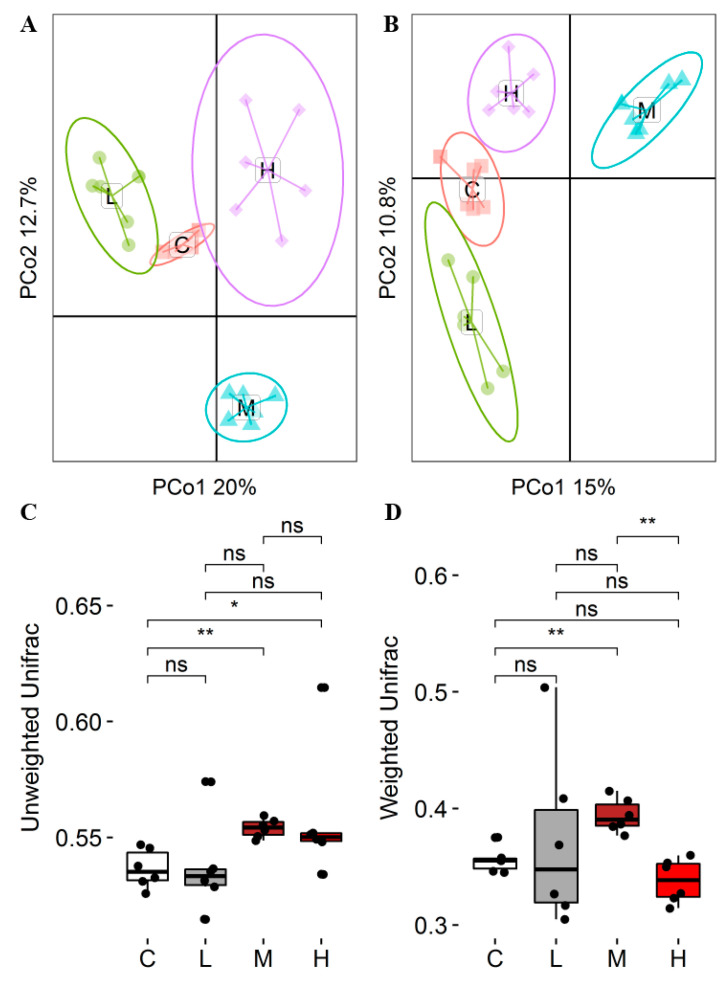
Beta diversity analysis of rumen microorganisms in the four groups. PCoA of rumen microbial composition based on unweighted (**A**) and weighted UniFrac (**B**). Unweighted (**C**) and weighted UniFrac (**D**) analysis box diagram. “ns” represents *p* > 0.05, “*” represents *p* < 0.05. “**” represents *p* < 0.05. C = CG, L = LG, M = MG, H = HG.

In this study, the observed species of MG were reduced by 8.05% compared to CG (*p* < 0.05), and the difference between the other test groups and CG was not significant (*p* > 0.05). The Shannon index of MG was reduced by 11.94% compared to CG (*p* < 0.05), and the difference between the other test groups and CG was not significant (*p* > 0.05). The Simpson index was not significantly different from CG (*p* > 0.05). The coverage of all groups was above 99%, indicating that the diversity of rumen microflora was relatively consistent and stable among individuals in the group (Table 7). PCoA analysis based on an unweighted UniFrac distance matrix showed that there was no significant difference between CG and LG (*p* > 0.05), but significant differences were observed between CG and MG, HG (*p* < 0.05). The PCoA analysis based on the weighted UniFrac distance matrix showed significant differences between CG and MG (*p* < 0.05) and significant differences between MG and LG (*p* < 0.05), indicating differences in the composition and structure of rumen microbiota among different groups (Figure 1).

**Figure 2:**
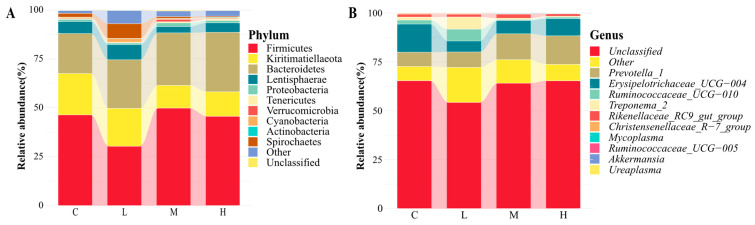
Flora composition of rumen microorganism at phylum (**A**) and genus (**B**) levels. C = CG, L = LG, M = MG, H = HG.

At the phylum level (Figure 2A), Firmicutes and Bacteroidetes had the highest abundance. The abundance of Lentisphaerae and Bacteroidetes in MG and HG was higher than in CG and LG (*p* < 0.05). Verrucomicrobia in MG was higher than in the other three groups (*p* < 0.05). Cyanobacteria, Proteobacteria, and Spirochaetae in LG were higher than in the other three groups (*p* < 0.05). At the phylum level (Figure 2B), *Prevotella 1* and *Erysipelotrichaceae UCG-004* were the dominant genera, and *Prevotella 1* in MG was significantly higher than in LG (*p* < 0.05), and there was no significant difference in the other genera (*p* > 0.05).

**Figure 3:**
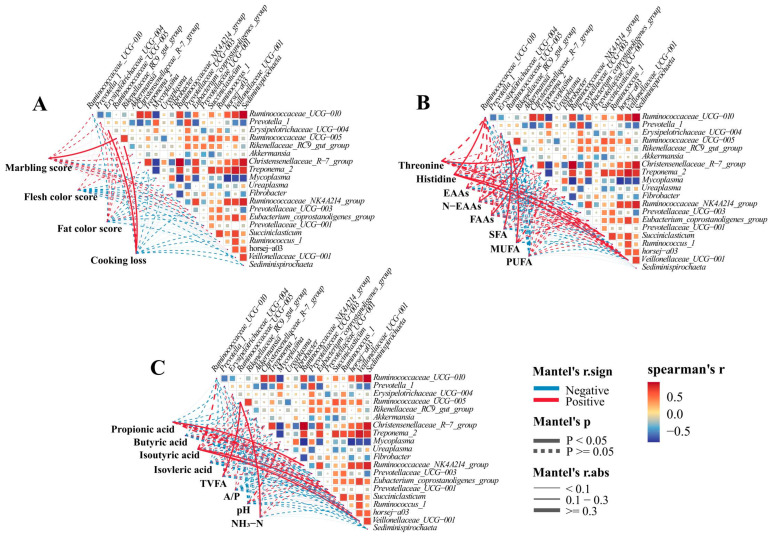
The correlation network heatmap between carcass quality (**A**), muscle amino acids and fatty acids (**B**), rumen fermentation (**C**), and the relative abundance of the top twenty genera is shown. The Spearman rank correlation coefficient is >0.5 or <−0.5. The red line represents a positive correlation coefficient, while the blue line represents a negative correlation coefficient. The actual situation of the straight line indicates significance, and the thicker the straight line, the greater the correlation. In the middle heat map, the red squares represent positive correlation, while the blue squares represent negative correlation. The size of the squares and the intensity of the colors reflect the strength of the correlation. In the legend on the right, different color ranges represent different correlation coefficients. EAAs: essential amino acid; N-EAAs: non-essential amino acids; FAA: flavor amino acids; SFA: saturated fatty acids; MUFA: monounsaturated fatty acids; PUFA: polyunsaturated fatty acids; A/P: acetic acid/propionic acid. The same is below.

To explore the main bacterial genera that affect carcass quality and rumen fermentation, relevant indicators and main bacterial genera were selected for mantel correlation testing of abundance (Figure 3). As shown in Figure 3A, for carcass quality, *Ruminococcaceae_UCG-005* showed a significant positive correlation with marbling score (r = 0.651, *p* < 0.05); *Erysipelotrichaceae_UCG-004* and *Rikenellaceae_RC9_gut_group* there was a significant positive correlation between group and cooking loss (r = 0.408, r = 0.347, *p* < 0.05). Regarding muscle amino acids and fatty acids (Figure 3B). *Akkermansia* showed a significant positive correlation with Threonine, N-EAAs, and FAAs (r = 0.547, r = 0.634, r = 0.601, *p* < 0.05); *Ruminococcaceae_UCG-010*, *Succinniclassicum*, *Veillonellaceae_UCG-001* showed a significant positive correlation with histidine (r = 0.403, r = 0.375, r = 0.365, *p* < 0.05); *Ruminococcaceae_UCG-005* showed a significant positive correlation with MUFA (r = 0.443, *p* < 0.05). In terms of rumen fermentation (Figure 3C), there was a significant positive correlation between the *Ruminococcaceae_NK4A214_group* and propionic acid (r = 0.326, *p* < 0.05). Additionally, *Succinniclassicum*, *horsej-a03*, and *Veillonellaceae_UCG-001* showed a significant positive correlation with isobutyric acid (r = 0.322, r = 0.291, r = 0.328, *p* < 0.05). Furthermore, *Ruminococcaceae_UCG-005* was significantly positively correlated with TVFA and NH_3_-N, respectively (r = 0.486, r = 0.513, *p* < 0.05).

### 3.5. Functional Prediction of the Rumen Microbiome

**Figure 4:**
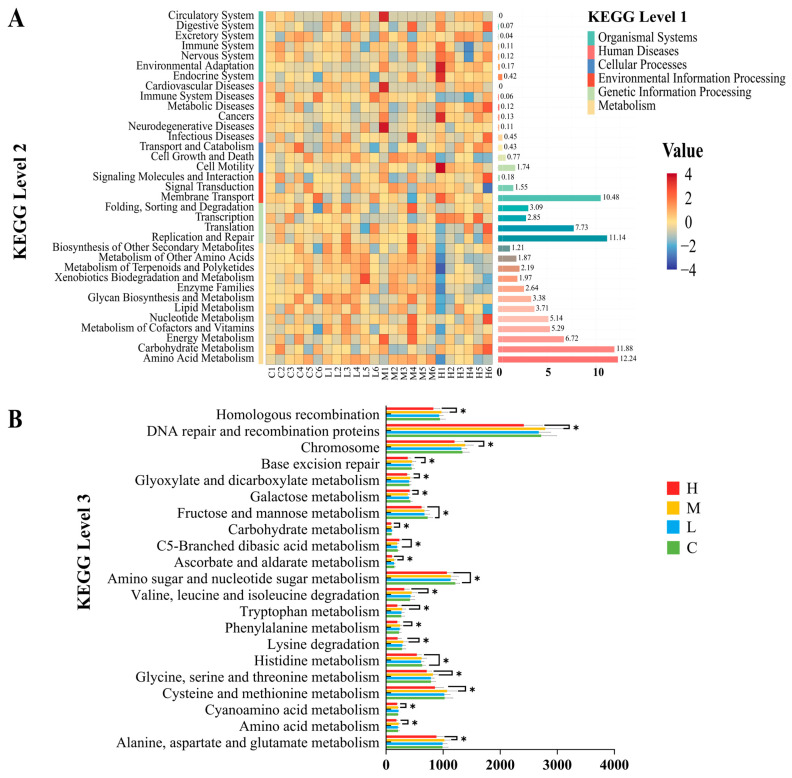
PICRUSt functional prediction KEGG Pathway L1, L2 hierarchical classification heatmap and bar chart (**A**); the color corresponds to the Z value calculated after normalizing the relative abundance of the function. The closer the color is to red, the greater the abundance; 4 groups Pathway L3 hierarchical metabolic pathway bar chart (**B**). “*” represents *p* < 0.05. C = CG, L = LG, M = MG, H = HG.

The functionality of rumen microbiota in experimental goat samples was predicted using the PICRUSt2 (v1.0) software. Pathway hierarchical classification statistics revealed that, at level 2, the main KEGG pathways in each group were primarily focused on amino acid metabolism (12.24%), carbohydrate metabolism (11.88%), replication and repair (11.14%), and membrane transport (10.48%) (Figure 4A). At level 3, significant differences (*p* < 0.05) were observed in the abundance of 21 functional genes related to metabolic pathways among the four groups (Figure 4B).

**Figure 5:**
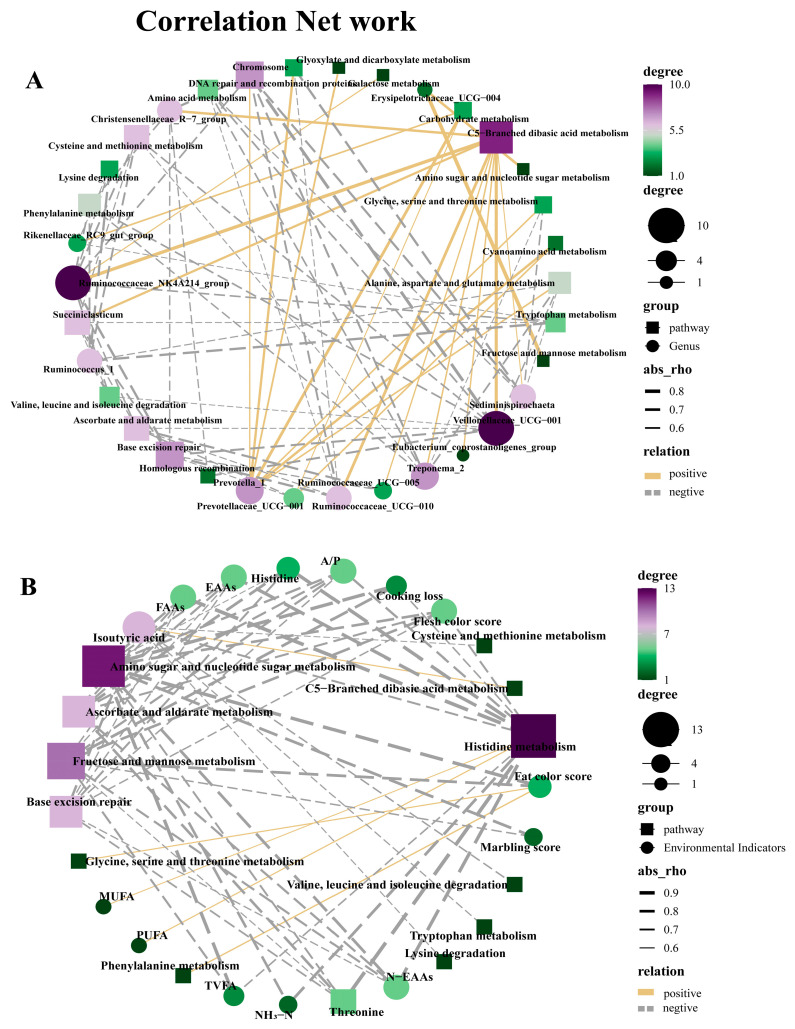
Analysis of the correlation network between KEGG Leve3 and carcass quality, rumen fermentation (**A**), and the top 20 microbial genera (**B**). The square in the figure represents the pathway, while the circle represents the genus or environmental indicator. Their size and color depth (green to purple) represent the degree of the relevant objects in the legend on the right. The purple and green nodes represent their centrality. The lines connected to nodes indicate significant correlations between species, and the thickness of the lines indicates the strength of the correlation. (|r| > 0.5, *p* < 0.05), gold and silver represent positive and negative correlations, respectively.

These pathways were further analyzed for their interaction networks with rumen microbiota, carcass quality, and rumen fermentation-related indicators. The analysis revealed that regarding carcass quality, the fat color score was positively correlated with glycine, serine and threonine metabolism and phenylalanine metabolism (r = 0.608, r = 0.629, *p* < 0.05); MUFA and PUFA were positively correlated with histidine metabolism (r = 0.587, r = 0.610, *p* < 0.05); isobutyric acid was positively correlated with C5-Branched dibasic acid metabolism (r = 0.608, *p* < 0.05) (Figure 5A). In terms of rumen microbiota and metabolic pathways, a total of 22 positive correlations were observed. *Prevotella 1* showed a strong positive correlation with seven metabolic pathways, including alanine, aspartate, and glutamate metabolism, cyanoamino acid metabolism, glycine, serine, and threonine metabolism, carbohydrate metabolism, glyoxylate, and dicarboxylate metabolism, and chromosome, DNA repair, and recombination proteins (r > 0.5, *p* < 0.05). Furthermore, C5-Branched dibasic acid metabolism exhibited positive correlations with nine bacteria, namely *Ruminococcaceae_UCG-010*, *Ruminococcaceae_UCG-005*, *Christensenellaceae_R-7_group*, *Treponema_2*, *Ruminococcaceae_NK4A214_group*, *Eubacterium_coprostanoligenes_group*, *Succiniclasticum*, *Veillonellaceae_UCG-001*, and *Sediminispirochaeta* (r > 0.5, *p* < 0.05) (Figure 5B).

## 4. Discussion

Sensory indicators of meat quality (marble score, meat color, fat color, etc.) and intrinsic indicators (cooking loss, pH, and shear force) are commonly used for evaluating meat quality [30]. Marble score can be used to measure the juiciness and tenderness of muscles, while meat color is the main sensory indicator that influences consumers’ purchasing decisions [31]. Although the correlation between muscle color and meat flavor is weak, it strongly affects consumer preferences [32]. The change in flesh color is mainly determined by the proportion of ferrimyoglobin (bright red), myoglobin (dark red), and metmyoglobin (gray, brown) in muscles [33]. The color of fat can reflect lipid deposition in muscles [34]. Previous studies have found that the supplementation of the spent mushroom substrates improves animal carcass quality [35]. This study reached the same conclusion that feeding SHMS to goats can improve meat quality and increase marbling, meat color score, and fat color score. Among them, the abundance of marble score in Chuanzhong black goats is relatively low. After adding SHMS to the diet, the marble scores of MG and HG were significantly higher than those of CG. It may be because *Hypsizygus marmoreus* is rich in natural antioxidants and characteristic bioactive compounds [36], and there are still residues in SHMS after mushroom harvest. On the other hand, lipid oxidation can alter the chemical properties of heme and cause myoglobin oxidation, resulting in the loss of brown color [37]. Antioxidants can inhibit the conversion of myoglobin in meat to metmyoglobin, protecting mutton from discoloration [38], thus affecting the grading of meat and fat color. This indicates that the sensory index of SHMS goat muscle is superior to that of the control group. As for intrinsic indicators, cooking loss is utilized to evaluate muscle water retention during cooking [39]. Different dietary supplements may produce variations in the tissue structure of mutton, leading to different changes in the cooking loss of longissimus dorsi [16], a trend similar to the results of this experiment. The pH value is a physical indicator of muscle acidity and alkalinity and is related to the degradation of glycogen and the release of lactic acid before and after slaughter [40]. It plays an important role in the biochemical processing after slaughter [41]. Shear force is used to describe the tenderness of meat, and it is directly influenced by muscle fibers and intramuscular fat [42]. In this study, there were no significant differences in the pH and shear force between the experimental groups, indicating that the intrinsic quality of muscles is less affected by the amount of SHMS added. Additionally, the hygiene standards for the experimental goat carcasses complied with national regulations, suggesting the feasibility of adding SHMS to the diet.

The composition of amino acids and fatty acids is closely related to the flavor of meat [43]. Therefore, this experiment evaluated the effect of adding SHMS to goat diets on the amino acid and fatty acid composition of the meat. Previous studies have shown that the addition of fertilized spent mushroom substrate from *Pleurotus eryngii* to Hu sheep can increase the amino acid content of lamb meat [17]. In line with the results of this experiment, the addition of SHMS significantly increased the content of histidine and tyrosine in goats. Moreover, with the increase in the added amount, the content of EAAs, N-EAAs, and FAAs showed an upward trend, indicating that SHMS can help to improve the nutritional value of lamb. The fishy taste of goat meat is one of the main factors affecting consumer consumption, primarily caused by volatile compounds produced from the oxidation of saturated fatty acids (SFAs) [44]. In this study, there was no significant difference in SFA levels among the groups, indicating that substituting 20–40% SHMS for roughage (silage corn and oat hay) in the fattening goat diet had little effect on the fishy smell of lamb meat. Furthermore, although the MG group exhibited relatively low levels of monounsaturated fatty acids (MUFAs) and polyunsaturated fatty acids (PUFAs), there was no significant difference in the fatty acid profile among the four groups. It is possible that certain bioactive components can influence the fatty acid composition of lamb, which needs to be further investigated.

On the other hand, the improved carcass quality of goats fed in this study may be attributed to the higher content of total volatile fatty acids (TVFAs) in the rumen. Rumen pH and volatile fatty acid (VFA) content are important indicators for evaluating ruminant fermentation [45]. VFA accounts for 80% of the total energy required by ruminants [46]. In this research, the addition of 30% SHMS (MG) significantly reduced the rumen pH, which may be related to changes in rumen VFAs. These VFAs are mainly produced by the fermentation of feed carbohydrates by rumen microorganisms and include short-chain fatty acids such as acetic acid, propionic acid, and butyric acid. Propionic acid in the rumen is a precursor of glucose synthesis, produced by gluconeogenesis in the liver, providing energy for the body [47]. Acetic acid and butyric acid are precursors for fat synthesis. The former is a product of fiber degradation and the primary carbon resource for the synthesis of milk fat and body fat [48]. The latter is converted into β-hydroxybutyric acid and participates in the citric acid cycle [49]. This study shows that the VFA content of MG (70.26 mmol/L) is significantly higher than CG (48.77 mmol/L). This may be attributed to the use of alkaline storage methods (5% CaO and 3% urea) in this experiment. The NH_3_-N produced by urea hydrolysis in the rumen stimulates the absorption of propionic acid by the rumen epithelium while providing a nitrogen source for cellulose-degrading bacteria. This process promotes cellulose degradation, leading to increased production of acetic acid and higher VFA content in the rumen [50]. On the other hand, a high content of propionic acid and the ratio of acetic acid to propionic acid indicate a higher energy utilization rate [51]. In this experiment, with the increase in SHMS addition, the acetic acid/propionic acid ratio gradually increased, and HG was significantly higher than CG. Additionally, NH_3_-N is an important product of rumen-fermented feed protein, endogenous protein, and non-protein nitrogen, providing a nitrogen source for rumen microorganisms to synthesize bacterial protein, with an effective concentration of 5–30 mg/100 mL [52]. In this experiment, the concentration of NH_3_-N ranged from 16.30 to 25.02 mg/100 mL, falling within the normal range and proving beneficial for microbial growth, and the NH3-N content of MG is the highest, exhibiting a trend of initially increasing and then decreasing. This trend may be attributed to the limitation of carbohydrate fermentation speed, which affects the animal’s utilization of urea [53,54]. Therefore, this experiment suggested that 30% SHMS was a suitable addition amount; at this time, the state of TVFA, pH, and NH_3_-N in the rumen was the best. In summary, based on the current research results, the supplementation of SHMS in the diet has a positive effect on promoting rumen fermentation and improving the carcass quality of goats. These results indicate that SHMS has nutritional value as ruminant feed.

Rumen microorganisms play a crucial role in the digestion, absorption, and metabolism of nutrients in ruminants [55]. This research demonstrated that the addition of SHMS resulted in changes in rumen α diversity, observed species, and Shannon index. The values of MG were significantly lower in comparison to CG. Additionally, the beta diversity analysis revealed that different levels of SHMS addition altered the rumen microbial community of goats. However, different diets did not alter the fact that Firmicutes and Bacteroidetes are the most abundant bacteria in the rumen of goats. Consistent with previous research results [56], this experiment identified Firmicutes and Bacteroidetes as the dominant phyla. Firmicutes play a key role as cellulose decomposers. Bacteroidetes is the primary decomposer of non-fiber plant polysaccharides and proteins in the rumen, and its relative abundance is correlated with the dietary NDF level [57,58]. This research revealed that there was no statistically significant difference in the relative abundance of Firmicutes among the four groups. However, the content of MG Bacteroidetes was significantly higher compared to CG and LG. At the genus level, these genera were not affected by the addition of SHMS to the diet. *Prevotella 1* rumen is the main dominant genus in the rumen, abundant in high-fiber diets, and plays a crucial role in the degradation of high molecular weight substances such as starch and protein [59]. The predominant bacteria at the genus level in the four groups of rumen content samples in this study was *Prevotella 1*, consistent with previous studies [60]. Among them, Prevotella exhibited the highest abundance in the MG group, which may be due to the fact that the MG after composite treatment contains more non-fibrous plant polysaccharides and non-protein nitrogen, and the C/N ratio is more suitable for the growth of *Prevotella 1*. Further research is needed to determine the specific reasons.

Rumen bacteria are closely related to animal carcass quality and rumen fermentation [61]. Therefore, we evaluated whether there is a correlation between bacterial genera, carcass quality, and rumen fermentation. The *Rikenellaceae_RC9_gut_group* and *Ruminococcaceae_UCG-005* play a crucial role in carbohydrate degradation, which is important for fermenting cellulose and other complex carbohydrates [62,63]. *Erysipelotrichaceae_UCG-004* can produce metabolites that enhance acids and reduce rumen pH [64]. In this investigation, *Ruminococcaceae_UCG-005* was positively correlated with marble score, TVFA, NH_3_-N, and MUFA; *Erysipelotrichaceae_UCG-004* and *Rikenellaceae_RC9_gut_group* were positively correlated with cooking loss, indicating that these bacterial genera may affect the carcass quality and rumen fermentation of goats by regulating carbohydrate degradation processes or producing metabolites. On the other hand, research has shown that *Ruminococcaceae_UCG-010*, *Ruminococcaceae_NK4A214_group*, *Succinniclassicum*, and *Veillonellaceae* can promote the degradation of cellulose and hemicellulose in animal rumen [65,66]. This study indicates a positive correlation between the *Ruminococcaceae_NK4A214_group* and propionic acid, as well as between *Ruminococcaceae_UCG-010* and Veillonellaceae with isobutyric acid, similar to previous research results [67]. This indicates a close correlation between rumen microbiota and VFA production. This indicates a close correlation between rumen microbiota and VFA production. These results indicate that adding SHMS to the diet can promote microbial growth and regulate rumen fermentation in goats. In addition, other studies have shown that *Alloprevotella* may play an important role in the fermentation of structural carbohydrates in the rumen of goats, thereby promoting energy absorption and affecting meat quality [68]. In research, it was also found that *Akkermansia* showed a significant positive correlation with threonine, N-EAAs, and FAAs, similar to a previous study showing a positive correlation between rumen microbiota and fatty acid production [16]. Overall, correlation analysis provides some reference for us to understand the relationships between rumen bacterial communities and carcass and rumen fermentation.

In addition, we also conducted KEGG pathway prediction and network interaction analysis between the KEGG pathway and bacterial genera, carcass quality, and rumen fermentation. It was found that at the second level, the KEGG pathways in each group were mainly concentrated in amino acid metabolism, carbohydrate metabolism, replication and repair, and membrane transport. There was no significant difference among the four groups in this experiment. However, at the third level, 21 pathways with significant differences were found, all of which showed the highest MG enrichment content and a significant decrease in HG. It is worth noting that carbohydrate metabolism and amino acid metabolism play an important role in the rumen [69]. Carbohydrates are one of the carbon sources for rumen bacteria, especially Bacteroidetes and Firmicutes, which can decompose complex carbohydrates with the help of digestive enzymes [70]. Previous studies have demonstrated that amino acids serve as one of the primary nitrogen sources for these bacteria, and the influence of amino acid metabolism pathways on bacterial protein synthesis and utilization is crucial [71]. It is speculated that rumen microorganisms may indirectly affect the deposition of metabolites through interactions with the host [72]. Previous studies have shown a close correlation between amino acid metabolism and meat quality [73]. This research also found a close correlation between fat color score, MUFA, PUFA, and amino acid metabolism (glycine, serine, threonine, phenylalanine, and histidine). In addition, the previous experimental results showed that with the addition of SHMS, the content of these indicators showed an increasing trend in each group, which was also demonstrated in KEGG L3 prediction. However, the difference is that at 40% of SHMS addition (HG), the enrichment of metabolic pathways is significantly reduced. Adding an appropriate proportion of SHMS to the diet can increase metabolic pathways, but excessive addition can reduce their enrichment. In the network diagram between KEGG and bacterial genera, it was observed that *Prevotella 1* showed a positive correlation with seven metabolic pathways. Previous studies have highlighted Prevotella as the predominant and early colonizer, occupying various ecological niches in the rumen [74]. These early-arriving species play a crucial role and have a long-term impact on the development of animal microbiota [75]. In addition, it has been demonstrated that C5-Branched chain amino acid metabolism is intrinsically linked to energy generation [76]. C5-Branched chain amino acids are converted into other metabolites through glycolysis in the rumen, thereby providing energy and nutrients for ruminants. The network graph in this experiment revealed a positive correlation between C5-Branched chain amino acid metabolism and nine bacterial genera, emphasizing the crucial role played by the microbial community in the rumen during this process. In summary, the addition of SHMS does indeed impact the rumen microbiota. Moreover, in the subsequent research, metabolites in the rumen can be examined through metabolomics to further investigate the potential mechanisms of adding SHMS to the diet on rumen bacteria, carcass quality, and rumen fermentation in Sichuan Black Mountain goats.

## 5. Conclusions

This study showed that adding SHMS to the diet changed the rumen microbial community structure of Chuanzhong black goats, had a positive impact on rumen fermentation, and ultimately improved goat carcass quality. In summary, the results of this study confirm the applicability of SHMS as a dietary component for goats. At the same time, fully utilizing abandoned resources can not only reduce the breeding cost of goats but also reduce environmental pollution.

## Figures and Tables

**Table 1:** Dietary formula and nutritional level (DM basis, %).

Ingredients	CG	LG	MG	HG
Diet composition/%				
Composite alkali storage mushroom bran	0.00	20.00	30.00	40.00
Silage corn	30.00	20.00	15.00	10.00
Oatmeal hay	15.70	12.05	10.57	7.92
Corn	26.39	28.81	28.00	27.34
Wheat bran	11.31	6.18	5.27	5.00
Cardamom	14.66	11.02	9.22	7.80
NaCl	0.22	0.22	0.22	0.22
Premix ^1^	1.72	1.72	1.72	1.72
Total	100.00	100.00	100.00	100.00
Nutritional level ^2^				
DMI (kg/d)	0.84	0.84	0.85	0.86
ME (KJ/d)	8.56	8.56	8.55	8.54
CP (g/d)	135.10	135.56	135.10	135.81
Ca (g/d)	4.00	4.00	4.00	4.00
P (g/d)	2.00	2.00	2.00	2.00

^1^ Premium: Each kilogram of premix contains 45 g of Na; Cl 55 g; Ca 330 g; P 160 g; Co 7 mg; Cu 1400 mg; I 27 mg; Fe 3200 mg; Mn 1000 mg; Se 35 mg; Zn 1000 mg; VA 780KIU; VE 10KIU. ^2^ Nutrient levels are calculated values. DMI: dry matter intake; ME: metabolizable energy; CP: crude protein; Ca: calcium; P: phosphorus.

**Table 2:** Effect of composite alkali storage mushroom bran on carcass quality of fattening goats.

Items	CG	LG	MG	HG	*p*-Value
Marbling score	1.00 ± 0.00 ^a^	1.00 ± 0.00 ^a^	2.00 ± 0.89 ^b^	1.50 ± 0.63 ^b^	0.038
Flesh color score	5.50 ± 0.55 ^a^	6.17 ± 0.41 ^b^	6.33 ± 0.52 ^b^	6.50 ± 0.55 ^b^	0.043
Fat color score	4.17 ± 0.41 ^a^	4.83 ± 0.41 ^b^	5.17 ± 0.41 ^b^	4.67 ± 0.52 ^ab^	0.032
pH value	5.85 ± 0.09	5.75 ± 0.07	5.80 ± 0.11	5.95 ± 0.15	0.218
Cooking loss/%	0.35 ± 0.02 ^a^	0.37 ± 0.05 ^ab^	0.40 ± 0.02 ^b^	0.38 ± 0.02 ^ab^	0.041
Shear force/N	55.85 ± 7.20	49.21 ± 7.28	60.67 ± 11.00	50.12 ± 11.05	0.446

^a,b^ Means with different lowercase superscripts in the same row differ significantly (*p* < 0.05).

**Table 3:** Effect of composite alkali storage mushroom bran on health index of finishing goats.

Items	CG	LG	MG	HG	Standard
Volatile salt base/(mg/100 g)	9.38	12.10	6.25	5.65	≤15
Hg/(mg/kg)	Undetected	Undetected	Undetected	Undetected	Not detectable
As/(mg/kg)	Undetected	Undetected	Undetected	Undetected	≤0.05
Cd/(mg/kg)	0.017	0.013	0.0036	0.0084	≤0.1
Pb/(mg/kg)	Undetected	Undetected	Undetected	0.046	≤0.2
Cr/(mg/kg)	0.077	0.064	0.066	0.055	≤0.1

Hg: hydrargyrum; AS: arsenic; Cd: cadmium; Pb: plumbum; Cr: chromium.

**Table 4:** Effect of composite alkali storage mushroom bran on the amino acid composition of fattening goat muscle.

Items/%	CG	LG	MG	HG	*p*-Value
Essential amino acid					
Threonine	0.99 ± 0.04	0.99 ± 0.02	1.08 ± 0.04	1.13 ± 0.13	0.107
Valerine	0.89 ± 0.03	0.90 ± 0.01	0.98 ± 0.03	1.01 ± 0.09	0.066
Methionine	0.44 ± 0.00	0.40 ± 0.01	0.56 ± 0.07	0.56 ± 0.16	0.119
Isoleucine	0.88 ± 0.02	0.90 ± 0.01	0.99 ± 0.05	1.00 ± 0.08	0.074
Leucine	1.61 ± 0.06	1.60 ± 0.03	1.78 ± 0.08	1.83 ± 0.18	0.058
Phenylalanine	0.83 ± 0.00	0.85 ± 0.01	0.95 ± 0.05	0.95 ± 0.08	0.064
Lysine	1.74 ± 0.05	1.75 ± 0.04	1.95 ± 0.14	1.97 ± 0.18	0.078
Histidine	0.88 ± 0.01 ^a^	0.85 ± 0.07 ^a^	0.98 ± 0.05 ^ab^	1.06 ± 0.07 ^b^	0.006
EAAs	8.26 ± 0.21	8.24 ± 0.20	9.27 ± 0.51	9.51 ± 0.97	0.068
Non-essential amino acids					
Aspartic acid	1.73 ± 0.01	1.70 ± 0.01	1.90 ± 0.09	1.96 ± 0.21	0.062
Serine	0.77 ± 0.02	0.79 ± 0.01	0.85 ± 0.06	0.88 ± 0.08	0.095
Glutamic acid	2.98 ± 0.03	3.02 ± 0.05	3.35 ± 0.19	3.40 ± 0.30	0.062
Glycine	0.88 ± 0.01	0.93 ± 0.08	0.91 ± 0.03	1.01 ± 0.06	0.075
Alanine	1.14 ± 0.02	1.16 ± 0.04	1.22 ± 0.06	1.29 ± 0.10	0.069
Cystine	0.38 ± 0.01	0.38 ± 0.00	0.43 ± 0.02	0.45 ± 0.06	0.061
Tyrosine	0.84 ± 0.01 ^a^	0.84 ± 0.01 ^a^	0.94 ± 0.04 ^ab^	0.98 ± 0.08 ^b^	0.011
Arginine	1.26 ± 0.03	1.28 ± 0.02	1.37 ± 0.08	1.40 ± 0.19	0.349
Proline	0.58 ± 0.03	0.61 ± 0.06	0.60 ± 0.04	0.64 ± 0.09	0.675
N-EAAs	10.56 ± 0.17	10.71 ± 0.25	11.57 ± 0.61	12.01 ± 1.17	0.088
FAAs	7.94 ± 0.08	8.04 ± 0.19	8.76 ± 0.44	9.06 ± 0.81	0.061

^a,b^ Means with different lowercase superscripts in the same row differ significantly (*p* < 0.05). EAAs: essential amino acid; N-EAAs: non-essential amino acids; FAA: flavor amino acids.

**Table 5:** Effect of composite alkali storage mushroom bran on intramuscular fatty acids in fattening goats.

Items/(g/100 g)	CG	LG	MG	HG	*p*-Value
C14:0	0.02 ± 0.01	0.03 ± 0.01	0.01 ± 0.00	0.02 ± 0.00	0.119
C16:0	0.29 ± 0.04	0.35 ± 0.10	0.06 ± 0.01	0.22 ± 0.01	0.139
C17:0	0.01 ± 0.01	0.02 ± 0.01	0.02 ± 0.01	0.01 ± 0.00	0.33
C18:0	0.19 ± 0.03	0.25 ± 0.06	0.04 ± 0.01	0.15 ± 0.01	0.208
C22:0	0.02 ± 0.01	0.02 ± 0.00	0.01 ± 0.00	0.01 ± 0.00	0.052
C16:1	0.02 ± 0.01	0.02 ± 0.01	0.03 ± 0.02	0.02 ± 0.00	0.229
C17:1	0.03 ± 0.01	0.04 ± 0.00	0.01 ± 0.00	0.02 ± 0.00	0.052
C24:1	0.02 ± 0.01	0.02 ± 0.01	0.02 ± 0.00	0.02 ± 0.01	0.330
C18:1n9	0.60 ± 0.01	0.81 ± 0.02	0.14 ± 0.02	0.48 ± 0.03	0.637
C18:2n6	0.18 ± 0.05	0.16 ± 0.02	0.13 ± 0.03	0.12 ± 0.02	0.546
C20:5n3	0.11 ± 0.01	0.10 ± 0.01	0.05 ± 0.02	0.07 ± 0.01	0.649
∑SFA	0.53 ± 0.10	0.67 ± 0.18	0.14 ± 0.03	0.41 ± 0.02	0.191
∑MUFA	0.67 ± 0.04	0.89 ± 0.14	0.20 ± 0.04	0.54 ± 0.04	0.693
∑PUFA	0.29 ± 0.06	0.26 ± 0.03	0.18 ± 0.05	0.19 ± 0.03	0.721

∑SFA: saturated fatty acids (sum of C14:0 + C16:0 + C17:0 + C18:0 + C22:0); ∑MUFA: monounsaturated fatty acids (sum of C16:1 + C17:1 + C24:1 + C18:1 n9); ∑PUFA: polyunsaturated fatty acids (C18:2 n6 + C20:5 n3).

**Table 6:** Effect of composite alkali storage mushroom bran on rumen fermentation parameters of goats.

Items	CG	LG	MG	HG	*p*-Value
Acetic acid/%	63.31 ± 2.47	63.22 ± 3.13	64.32 ± 3.04	62.02 ± 0.08	0.742
Propionic acid/%	19.62 ± 2.58 ^b^	14.71 ± 2.42 ^ab^	14.75 ± 2.06 ^ab^	12.78 ± 3.42 ^a^	0.046
Butyric acid/%	1.79 ± 0.00 ^a^	4.88 ± 0.69 ^b^	2.53 ± 1.12 ^a^	2.78 ± 0.41 ^a^	0.003
Isobutyric acid/%	11.29 ± 0.58 ^a^	8.79 ± 1.45 ^ab^	13.51 ± 3.32 ^ab^	16.86 ± 2.74 ^b^	0.014
Isovaleric acid/%	2.13 ± 0.35 ^a^	7.89 ± 1.10 ^b^	3.91 ± 1.60 ^a^	4.48 ± 0.54 ^a^	0.001
Valeric acid/%	1.87 ± 1.04	1.78 ± 0.69	0.99 ± 0.34	1.09 ± 0.20	0.297
TVFA/mmol/L	48.77 ± 1.86 ^a^	53.34 ± 6.89 ^a^	70.26 ± 5.47 ^b^	56.26 ± 8.34 ^a^	0.013
Acetic acid/Propionic acid	3.26 ± 0.56 ^a^	4.38 ± 0.79 ^ab^	4.41 ± 0.79 ^ab^	5.03 ± 0.94 ^b^	0.019
pH value	7.05 ± 0.16 ^b^	7.03 ± 0.12 ^b^	6.53 ± 0.34 ^a^	6.87 ± 0.16 ^b^	0.037
NH_3_-N/(mg/100 mL)	16.30 ± 0.88 ^a^	16.36 ± 2.78 ^a^	25.02 ± 2.03 ^c^	19.64 ± 2.20 ^b^	0.018

^a,b,c^ Means with different lowercase superscripts in the same row differ significantly (*p* < 0.05). TVFA: total volatile fatty acids.

**Table 7:** Effect of composite alkali storage mushroom bran on alpha diversity of rumen microorganisms.

Items	CG	LG	MG	HG	*p*-Value
Observed species	1376.43 ± 42.98 ^b^	1354.37 ± 93.58 ^ab^	1265.62 ± 19.75 ^a^	1394.55 ± 117.64 ^ab^	0.026
Shannon index	6.45 ± 0.54 ^b^	6.73 ± 1.21 ^b^	5.68 ± 0.25 ^a^	6.04 ± 0.98 ^ab^	0.047
Simpson index	0.94 ± 0.02 ^ab^	0.96 ± 0.01 ^b^	0.91 ± 0.01 ^a^	0.93 ± 0.04 ^ab^	0.017
Coverage/%	99.23	99.45	99.41	99.25	/

^a,b^ Means with different lowercase superscripts in the same row differ significantly (*p* < 0.05).

## Data Availability

These data are contained in the article.

## Supplementary Materials

The following supporting information can be downloaded at: https://www.mdpi.com/article/10.3390/ani14010166/s1, Table S1: *Hypsizygus marmoreus* medium and the nutritional components of the mushroom bran; Table S2: Report on Pesticide Residues, Heavy Metal Residues and Aflatoxin Residues in Pleurotus ostreatus.
