# Supplementary material for: The Effects of Composite Alkali-Stored Spent Hypsizygus marmoreus Substrate on Carcass Quality, Rumen Fermentation, and Rumen Microbial Diversity in Goats
Source: Animals (Basel). 2024 Jan 4;14(1):166. doi: 10.3390/ani14010166 (PMC10778354; doi:10.3390/ani14010166)
Supplement: Supplementary file 1 [file animals-14-00166-s001.zip › animals-2766847-supplementary.pdf]

The Effects of Composite Alkali-Stored Spent *Hypsizygus marmoreus* Substrate (SHMS) on Carcass Quality, Rumen Fermentation, and Rumen Microbial Diversity in Goats

Supplementary Materials:

Table S1. *Hypsizygus marmoreus* medium and the nutritional components of the mushroom bran (DM basis, %) .

Medium formulation / %		Nutrient composition	
Cottonseed hull	50.00	Dry matter	94.85
Corncob	10.00	Ether extract	1.82
Wheat bran	10.00	Crude protein	13.16
Bagasse	10.00	Acid detergent fiber	41.84
Sawdust	10.00	Neutral detergent fiber	54.75
Soybean meal	5.00	Acid detergent lignin	17.26
Corn	5.00		
Total	100.00		

Table S2. Report on Pesticide Residues, Heavy Metal Residues and Aflatoxin Residues in Pleurotus ostreatus

Items	Results
$C_9H_9N_3O_2$/(mg/kg)	Undetected（＜0.1）
$C_{22}H_{22}NO_3$/(mg/kg)	Undetected（＜0.1）
$C_2H_8NO_2PS$/(mg/kg)	Undetected（＜0.1）
$C_4H_7Cl_2O_4P$/(mg/kg)	Undetected（＜0.1）
$C_4H_8Cl_3O_4P$ /(mg/kg)	Undetected（＜0.1）
$C_6H_6Cl_6$/(mg/kg)	Undetected（＜0.1）
$C_{13}H_9Cl_{15}$/(mg/kg)	Undetected（＜0.1）
Pb/(mg/kg)	Undetected
As/(mg/kg)	Undetected
Hg/(mg/kg)	Undetected
Cd/(mg/kg)	Undetected
Cr/(mg/kg)	Undetected
B_1/(μg/kg)	Undetected
